# Proteolytic Profiling of Streptococcal Pyrogenic Exotoxin B (SpeB) by Complementary HPLC-MS Approaches

**DOI:** 10.3390/ijms23010412

**Published:** 2021-12-30

**Authors:** Constantin Blöchl, Christoph Holzner, Michela Luciano, Renate Bauer, Jutta Horejs-Hoeck, Ulrich Eckhard, Hans Brandstetter, Christian G. Huber

**Affiliations:** 1 Bioanalytical Research Labs, Department of Biosciences, University of Salzburg, Hellbrunner Strasse 34, 5020 Salzburg, Austria; constantin.bloechl@sbg.ac.at; 2 Structural Biology, Department of Biosciences, University of Salzburg, Hellbrunner Strasse 34, 5020 Salzburg, Austria; christoph.holzner@sbg.ac.at (C.H.); ueccri@ibmb.csic.es (U.E.); johann.brandstetter@plus.ac.at (H.B.); 3 Molecular Immunology & Signal Transduction, Department of Biosciences, University of Salzburg, Hellbrunner Strasse 34, 5020 Salzburg, Austria; michela.luciano@plus.ac.at (M.L.); renate.bauer@plus.ac.at (R.B.); jutta.horejs-hoeck@plus.ac.at (J.H.-H.); 4 Cancer Cluster Salzburg, Department of Biosciences, University of Salzburg, Hellbrunner Strasse 34, 5020 Salzburg, Austria; 5 Department of Structural Biology, Molecular Biology Institute of Barcelona, CSIC, Barcelona Science Park, Baldiri Reixac, 15-21, 08028 Barcelona, Spain

**Keywords:** streptococcal cysteine protease, SCP, streptopain, protease degradomics, HUNTER, N-terminomics, positional proteomics, sequence specificity, sequence logo, IgG subclasses

## Abstract

Streptococcal pyrogenic exotoxin B (SpeB) is a cysteine protease expressed during group A streptococcal infection that represents a major virulence factor. Although subject to several studies, its role during infection is still under debate, and its proteolytic properties remain insufficiently characterized. Here, we revisited this protease through a set of complementary approaches relying on state of-the-art HPLC-MS methods. After conceiving an efficient protocol to recombinantly express SpeB, the zymogen of the protease and its activation were characterized. Employing proteome-derived peptide libraries, a strong preference for hydrophobic and aromatic residues at P2 alongside negatively charged amino acids at P3′ to P6′ was revealed. To identify relevant in vivo substrates, native proteins were obtained from monocytic secretome and plasma to assess their cleavage under physiological conditions. Besides corroborating our findings concerning specificity, more than 200 cleaved proteins were identified, including proteins of the extracellular matrix, proteins of the immune system, and proteins involved in inflammation. Finally, the cleavage of IgG subclasses was studied in detail. This study precisely depicts the proteolytic properties of SpeB and provides a library of potential host substrates, including their exact cleavage positions, as a valuable source for further research to unravel the role of SpeB during streptococcal infection.

## 1. Introduction

*Streptococcus pyogenes*, or group A streptococcus (GAS), is an exclusively human pathogen responsible for a number of mild and severe pathological conditions, including necrotizing fasciitis, toxic shock syndrome, and rheumatic fever [1]. The diversity of diseases caused by this pathogen ranges from toxin-mediated pathologies (over immune-mediated ones) to those resulting from the direct infection. Despite improvements in the number of fatalities of GAS infections due to the increased availability of antibiotics as well as socio-economic reasons, GAS infections remain a severe illness in developing countries and cause several hundred thousand deaths every year [2]. Moreover, with the emergence of novel strains, GAS infections may continue posing a threat to health in both developed as well as developing countries [3]. 

One of GAS’s major virulence factors is streptococcal pyrogenic exotoxin B (SpeB), also known as streptopain or streptococcal cysteine protease (SCP), a C10 family cysteine protease. The protease is conserved in virtually all GAS strains [4], and it was shown that the pathogen is dependent on its expression to cause disease [5]. Like many proteases, SpeB is secreted as a zymogen termed SpeBz, which may be activated autocatalytically or by other proteases into the active and mature dimeric SpeBm [6,7,8]. While the early phase of GAS infection may be characterized by a generally reduced proteolytic activity, SpeB is thought to be expressed at a later stage of infection under nutrient-restricted conditions to facilitate bacterial evasion into the surrounding tissue [9]. The complex and tight regulation of SpeB activity by GAS is thoroughly reviewed by Carroll et al. [10].

So far, knowledge about the substrate specificity of SpeB has been mainly gathered by characterization of the autocatalytic processing of SpeBz into SpeBm and by in vitro characterization employing fluorescently labeled peptides [7,11,12,13]. To our knowledge, nine different autocatalytically cleaved sites have been reported in the pro-domain of SpeB [7,11,12]. Relying on this rather limited set of cleavage sites and their surrounding amino acids, a preference for hydrophobic residues at the P2 site (second amino acid N-terminal to the scissile bond) was suggested [10]. In line with these observations, one early report describes cleavage sites of similar nature in human insulin [14]. Moreover, inferred from the sites of autocatalysis, positively charged residues may be preferred at the P1 site. In addition to these initial observations of substrate specificity, Nomizu et al. studied SpeBm activity on terminally labeled peptides of four to eight amino acids in length in an intramolecular quenched fluorescence assay [13]. Using this approach, they were able to confirm the preference of hydrophobic residues at the P2 site and proposed the need for an occupied P3 site. Intriguingly, a high increase in activity was reported if isoleucine was present in the P2 site rather than the structural isomer leucine. Besides those studies characterizing peptide substrates, several reports identified protein substrates of both human as well as streptococcal origin in in vitro and in vivo scenarios. Reported substrates include but are not limited to proteins of the extracellular matrix, e.g., fibrinogen and kininogen; proteins involved in the host’s immune response, e.g., pre-interleukin 1β and complement C3; and streptococcal proteins, e.g., M protein and C5a peptidase, which are extensively reviewed [9,10,15,16]. More controversially discussed is the cleavage of human IgGs. Although several studies reported the cleavage of human IgG1 at a specific site in the hinge region [17,18,19] and some proposed implications for host immune modulation [20,21], a recent publication states that in vivo human IgG may not be a target of SpeB [22]. Notably, under reducing conditions, SpeB has been used to generate Fc/2 subunits (C_H_2 and C_H_3 antibody domains) and Fd subunits (V_H_ and C_H_1 domains) from monoclonal human IgG1 antibodies [23], as well as from polyclonal murine IgGs [24].

Over the last two decades, high-performance liquid chromatography hyphenated to mass spectrometry (HPLC-MS) has emerged as a versatile and powerful tool to study proteins and peptides as substrates of proteases [25,26,27,28]. The most comprehensive approaches are based on peptide libraries that are incubated with the protease of interest and, eventually, subjected to HPLC-MS analysis [29,30]. Those methods that obtain a peptide library from proteomes are—based on the initial publication—commonly referred to as proteome-derived identification of protease cleavage sites (PICS) and come in many different varieties [30,31,32]. Whilst these approaches are a powerful tool to reveal substrate specificity and readily identify hundreds of cleavage sites in vitro, these methods neglect the accessibility of potential cleavage sites in intact proteins and are not capable of identifying novel in vivo targets. This bottleneck is overcome by approaches such as terminal amine isotopic labeling of substrates (TAILS), which are based on the incubation of intact protein substrates with the protease of interest, or even directly investigating biological samples [33]. Additionally, such N-terminomics approaches come in several varieties differing by their ways to deplete interfering N-termini, e.g., the HUNTER approach [34], or how to perform isotopic labeling [35]. To overcome some limitations of approaches based on the analysis of peptides, i.e., bottom-up proteomics, HPLC-MS techniques based on larger protein subunit analysis, e.g., middle-up/down and top-down, are employed for in-depth protease profiling [36,37].

In summary, the biological role of SpeB in disease is still under discussion, and some of its targets remain controversial. Although reasonable efforts have been undertaken to study its molecular regulation in GAS, its biochemical and structural properties, and its role in disease, its proteolytic properties remain insufficiently characterized, and some of its native substrates may have stayed undiscovered. In this study, we aimed to decipher the proteolytic properties and shed light on the substrate recognition of SpeB through a set of state-of-the-art HPLC-MS approaches. Based on complementary experimental approaches, we defined proteolytic targets at the peptide and protein levels to gain information on in vivo and in vitro cleavage properties of this protease in an unbiased manner. After characterization of recombinantly expressed SpeBz and its activation into mature SpeBm, we initially profiled its specificity towards peptide substrates. To this end, we performed PICS analysis of two complementary peptide libraries obtained from *E. coli* whole-cell lysates. Next, to mimic an in vivo scenario of infection, we employed N-terminomics based on the HUNTER protocol [34] for intact proteins, derived from the monocytic secretome and plasma samples. Eventually, we focused on human immunoglobulin G subclasses as model proteins with, although controversially discussed, potential relevance to the pathology of GAS. Taking the different kinds of substrates investigated together, our results display SpeB as an active cysteine protease, cleaving a broad range of host substrates potentially relevant during GAS infection with a distinct amino acid specificity clearly portrayed by our methods.

## 2. Results

### 2.1. Characterization of Recombinant SpeBm and SpeBz

**Figure 1:**
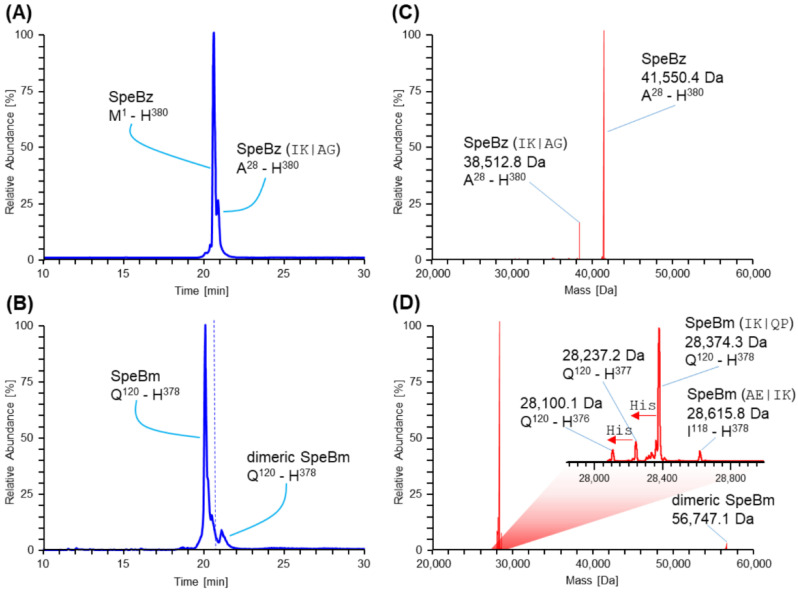
Characterization of recombinant SpeBz and SpeBm by means of HPLC-MS. Panel (**A**) and (**B**) show total ion current chromatograms (TICCs; blue) of MMTS-inhibited zymogenic SpeBz and activated SpeBm, respectively. The chromatographic peaks are labeled according to their deconvoluted masses (red) as determined by hyphenated mass spectrometric analysis (panels (**C**) and (**D**)). Depicted amino acid sequence numbers of protein species correspond to Figure S1D. Of note, residue Q_120_ corresponds to Q_146_ in the UniProt entry P0C0J0. This offset originates from construct design, lacking the signal peptide. To ease the comparison of the two chromatograms, a dashed blue line shows the retention time of SpeBz observed in panel (**A**) in panel (**B**). To distinguish different SpeB species, the specific cleavage site is listed in brackets (**C**,**D**). Mass shifts of SpeBm caused by degradation of the His-tag are indicated with red arrows and “His”.

SpeBz was recombinantly produced containing a C-terminal His6-tag and purified similarly to published protocols [38,39]. However, several amendments were made to optimize yield and specific activity, as stated earlier. To determine its properties and purity, SpeBz was inhibited immediately after purification by the addition of MMTS, reversibly blocking the active site cysteine, and subsequently analyzed by SDS-PAGE and HPLC-MS (Figure 1 and Figure S1). The amino acid numerations are listed in Figure S1D. Based on its molecular mass, we found the main species to be the uncleaved zymogen of 41,550 Da, accompanied by a minor species (38,513 Da) already cleaved at one of the distal sites reported in the literature (IK_27_|AG) [11]. Further, neither SDS-PAGE nor HPLC-MS analyses showed signs of protein impurities in our preparations. SpeBz could be efficiently transformed into its mature form by incubation at 37 °C for 30 min, as depicted in Figure 1 and Figure S1B. As expected, the main variant of SpeBm was fully processed by cleavage at the IK_119_|QP motive into the 28,374 Da mature protease. In addition, we observed the artificial hexahistidine tag to be sequentially digested off of the mature form (Figure 1D). An additional species of the protease (28,616 Da) was observed at around 5% relative abundance which still comprised two additional amino acids on its N-terminus due to incomplete proteolytic processing. Notably, we detected dimerized SpeBm in the HPLC-MS analyses after proteolytic activation. Employing azocasein digestion, we found a specific activity of approx. 12,500 U∙mg^−1^ under reducing conditions (Figure S1C and Table S2).

### 2.2. Proteome-Derived Identification of Protease Cleavage Sites (PICS)

**Figure 2:**
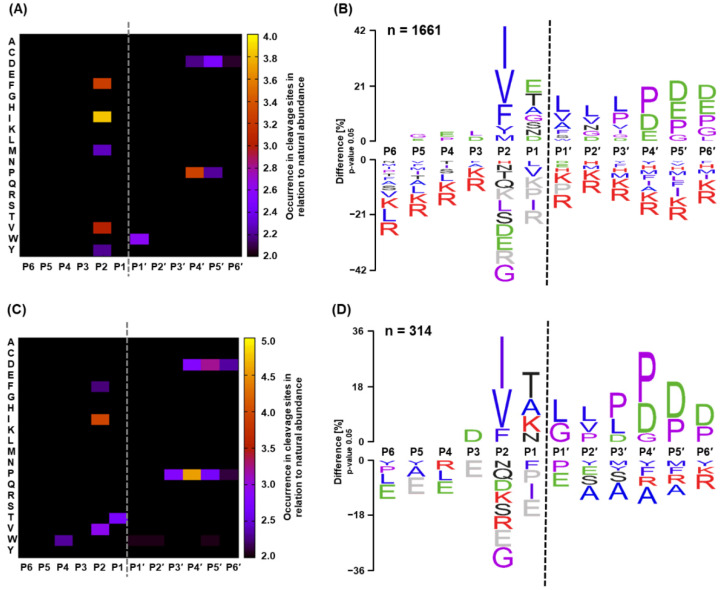
Identification of cleavage sites of SpeBm in peptide substrates derived from the *E. coli* proteome by PICS analysis. A cumulative number of 1975 unique cleavage events were determined in trypsin- (**A**,**B**) and GluC-derived (**C**,**D**) peptide libraries employing stable isotope labeling and HPLC-MS analysis. Heatmaps depict the fold-change in occurrence of specific amino acids at certain positions in the substrate in relation to their natural abundance using either (**A**) trypsin or (**C**) GluC as library protease. Sequence logos (**B**,**D**) demonstrate the difference in percent of amino acids at specific positions in prime and non-prime sites as obtained by the IceLogo algorithm. Data are based on (**C**) a tryptic or (**D**) a GluC-derived peptide library. The color code represents the physico-chemical properties of amino acids: hydrophobic residues (blue), positively charged residues (red), negatively charged residues (green), hydrophilic residues (black), and others (violet). Amino acids that were not found in any of the determined peptide substrates at a specific position are depicted in gray.

Next, the proteolytic properties of SpeBm were explored by the cleavage of peptide libraries created from the entire *E. coli* proteome. Specifically, cleavage specificity profiles were generated relying on published protocols combining proteome-derived peptide libraries and quantitative proteomics based on stable isotope labeling [31]. In this study, *E. coli*-derived proteins were digested with two library proteases that have well-documented specificities, namely trypsin and GluC. To ensure good quality of the data, the workflow was optimized and tested using GluC as test protease and trypsin as library protease (Figure S2). Subsequently, SpeBm was incubated with the two complementary peptide libraries, supplemented with reducing agents, over a timescale of three hours. Concerning the tryptic peptide library, a total of 6954 unique peptide sequences were identified, of which an astonishing 1661 peptide substrates were specifically cleaved by SpeBm. To complement the tryptic library, SpeBm cleavage specificity was additionally verified with a GluC-derived peptide library. Here, 6302 unique peptides were identified, of which 314 were proteolytically digested by SpeBm. For both cases, C-terminal fragments of cleaved peptides were used as input for the web tool CLIP-PICS, which determined the missing non-prime sites of the scissile bond via database lookup [40]. Based on the retrieved data, cleavage specificity was visualized in a heat map and a sequence logo for the tryptic peptide library (Figure 2A,B) and the GluC-derived peptide library (Figure 2C,D). Sequence specificity profiles obtained from the two independent libraries were highly consistent, apart from the differences introduced by the respective library protease. Taking the data together, SpeBm shows a prominent affinity for hydrophobic amino acids, i.e., isoleucine, valine, phenylalanine, and tyrosine, at the P2 site. However, leucine, which exhibits similar hydrophobicity, did not show any enrichment at the P2 site. Despite its very high prevalence in the *E. coli* proteome, leucine at the P2 site was only present in 5.9% (tryptic library) and 8.0% (GluC library) of the cleaved peptides analyzed (Tables S3 and S4). Astoundingly, SpeBm has a notable preference for negatively charged residues, i.e., glutamic and aspartic acid, at the substrate sites P4′ to P6′ and moderately pronounced affinity for hydrophobic residues, i.e., leucine and valine, at P1′ to P3′. Intriguingly, proline at either the P1 or P1′ position completely abrogated the proteolysis of peptide substrates, as none of the combined 1975 identified peptide substrates carried this amino acid at P1, and only a single proline was found at the P1′ position in these peptide substrates (Tables S3 and S4). Prolines, however, were usually located at the P3′ to P6′ positions, with the most prominent enrichment at P4′. Affinity for hydrophilic residues was mainly observed at the P1 site. Moreover, SpeBm showed little preference for any specific class of amino acids at the non-prime sites P6 to P3.

### 2.3. Proteolytic Processing of Intact Proteins by N-Terminomics

**Figure 3:**
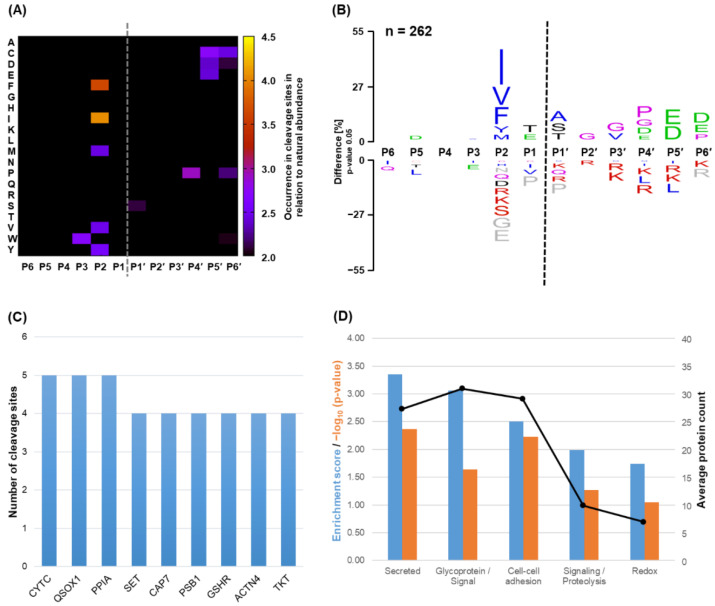
Identification of cleavage sites in intact proteins derived from the monocytic secretome. A total of 262 unique cleavage sites were identified by the N-terminomics workflow, combining cleavages identified after two individual experiments employing subsequent tryptic and GluC digestion, respectively. Based on this combined data, the heatmap in panel (**A**) depicts the fold-change in occurrence of specific amino acids at certain positions in the substrate in relation to their natural abundance. (**B**) Respective sequence logo demonstrating the difference in percent of amino acids at specific positions in prime and non-prime sites ranging from P6 to P6′. The amino acid color code is defined in the caption of Figure 2. (**C**) Top 9 proteins (UniProt abbreviations) in the secretome of monocytes ranked after the number of SpeB cleavage sites identified. (**D**) Top 5 enriched annotation clusters of proteins that were substrate to SpeB proteolysis based on enrichment analysis. The blue bar chart depicts the enrichment score, whereas the orange bars indicate the average negative log_10_ *p*-value. The interpolated points indicate the average number of proteins involved in the respective annotation cluster that were found to be proteolytically processed.

Since the processing of a protein substrate by a specific protease is not only governed by the amino acid sequence but also dependent on its accessibility, we performed an exploratory approach to identify potential substrates in their native conformation and under physiological conditions. Based on published highly optimized protocols [33,34], the proteolytic processing of intact proteins obtained from monocytic secretome, i.e., from the MOLM-13 cell line, and proteins from plasma samples were investigated. Digestion of these proteins was conducted under physiological conditions, i.e., at neutral pH, 37 °C, and without the addition of reducing agents. All identified novel N-termini and the corresponding C-terminal part of the cleavage sites, as well as the respective detected peptides for both experiments, are listed in Table S5. Initially, we assessed the cleavage of proteins secreted from the monocytic cell line MOLM-13 (Figure 3). Combining the two individual complementary experiments employing subsequent trypsin (Figure S3A,B) and GluC digestion (Figure S3C,D), respectively, 262 unique cleavage events and thus protein neo-N-termini were reported in 180 individual proteins, as specified in detail in Table S6. The specificity profile observed earlier for peptide substrates was corroborated by the obtained N-terminomics data. Consistently, a prominent preference for the hydrophobic residues isoleucine, valine, phenylalanine, and tyrosine was found, as illustrated in the respective heatmap (Figure 3A) and the corresponding sequence logo (Figure 3B). Again, proline at either side of the scissile bond efficiently inhibited proteolysis, as this amino acid was found in none of the cleaved substrates. Moreover, substantiating our results from PICS analysis, negatively charged residues were found enriched at the P4′ to P6′ sites. Interestingly, cysteine residues slightly emerged at the primed sites P5′ to P6′, as indicated by violet color in the heatmap. As observed in the PICS data, no specificity for any amino acid was found in the non-prime sites P6 to P3. To gain an overview of these protein substrates, the top nine most frequently cleaved proteins were listed (Figure 3C), with cystatin C (CYTC), a cysteine protease inhibitor; QSOX1, a cysteine oxidase; and PPIA, which is a proline isomerase, comprising the most identified cleavage sites. In addition to frequently cleaved proteins, we performed a functional annotation clustering identifying enriched molecular functions of those proteins that were found to be processed by SpeB, using all identified proteins in that secretome sample as a background dataset. In Figure 3D, we show the five most enriched clusters based on their enrichment score. Not surprisingly, we found many cleaved proteins that were categorized as “secreted” to be cleaved by SpeB. However, we additionally identified two clusters to be cleaved which comprise many proteins with reported signaling capacities. Furthermore, proteins controlling the redox state of cells and those involved in cellular adhesion appear to be likely targets of SpeB.

**Figure 4:**
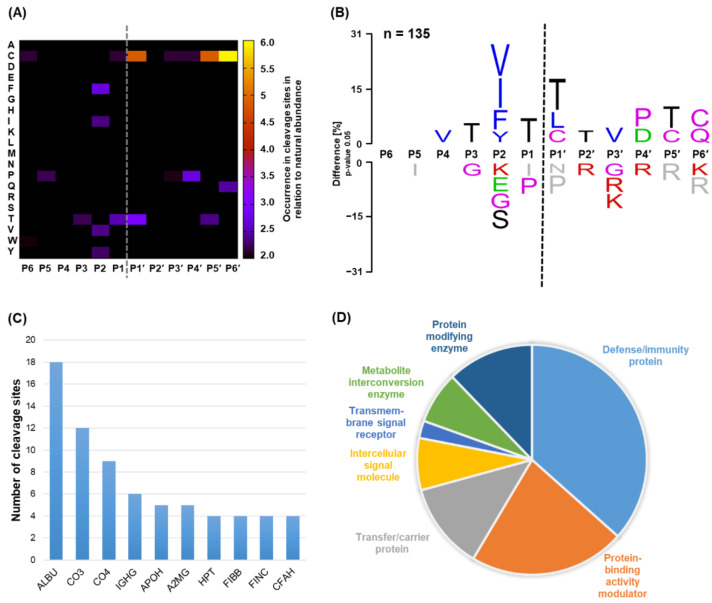
Identification of cleavage sites in intact proteins derived from plasma samples. A total of 135 cleavage sites were consistently identified by the N-terminomics workflow in plasma samples of two individual donors. Analysis was conducted based on cleavage sites that were combined from peptides identified after subsequent tryptic and GluC digestion, respectively. Based on these data, the heatmap in panel (**A**) depicts the fold-change in occurrence of specific amino acids at certain positions in the substrate in relation to their natural abundance. (**B**) Respective sequence logo demonstrating the difference in percent of amino acids at specific positions in prime and non-prime sites ranging from P6 to P6′. The amino acid color code is defined in the caption of Figure 2. (**C**) Top 10 proteins (UniProt abbreviations) in plasma ranked after the number of SpeB cleavage sites identified. (**D**) Overview of molecular functions terms (gene ontology) assigned to proteins that were cleaved by SpeB, illustrated by a pie chart.

To further expand our understanding of SpeB protein substrates, we applied the N-terminomics workflow to human plasma samples, combining experiments based on subsequent tryptic (Figure S4A,B) or GluC digestion for peptide identification (Figure S4C,D). Cleavage sites were only considered in further analyses if the respective peptide was identified in both independently performed experiments and using plasma samples from two different donors. Combining the data from tryptic and GluC digestion, 135 unique cleavage events were characterized in 53 proteins, as listed in Table S7. As described in all previous experiments, we observed a preference for isoleucine, valine, phenylalanine, and tyrosine at the P2 site (Figure 4A,B). In addition to those features already identified in the preceding PICS and N-terminomics experiments, the emergence of cysteine residues at primed sites stands out. In particular, at P5′ and P6′ but also at P1´, cysteine residues were enriched pronouncedly. Besides the specificity profile, we investigated those proteins that comprise the most identified cleavage sites and depicted the top 10 of these proteins. Besides albumin (ALBU), we identified the cleavage of several immunologically relevant proteins, e.g., proteins of the complement system (C3 (CO3), C4 (CO4), and factor H (CFAH)), alpha-2-macroglobulin (A2MG), and IgG proteins (e.g., IGHG). An overview of the digested proteins assigned to their respective protein classes is depicted in Figure 4D. Notably, we observed a large fraction of digested proteins to be involved in the defense and immune response.

### 2.4. Immunoglobulin G Subclasses as Substrates of SpeBm

**Figure 5:**
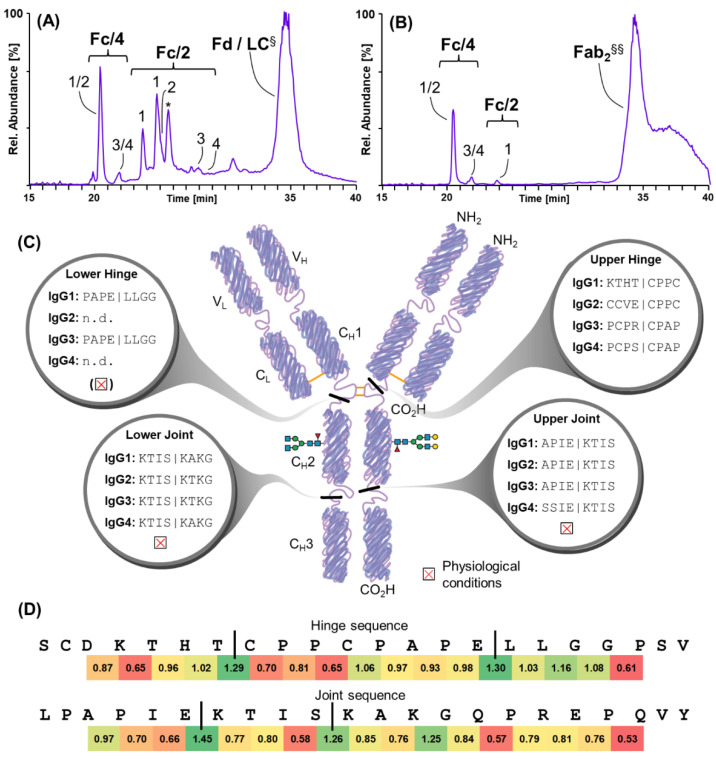
Proteolysis of IgG subclasses by SpeBm. Total ion chromatograms of human polyclonal IgG digested by SpeBm under (**A**) reducing or (**B**) physiological conditions. Main cleavage products are annotated: fragment crystallizable (Fc) and fragment antigen-binding (Fab), as well as their derivatives. Numbers denote the corresponding subclasses from which these subunits originate. Single peaks may be annotated by multiple numbers, as proteolytic products from different subclasses may co-elute. The asterisk (*) denotes a chromatographic peak corresponding to an incompletely alkylated IgG1 subunit cleaved at KTHT|CPPC. ^§^ Putative chromatographic peak of the Fd subunit and the light chain (LC). ^§§^ Putative chromatographic peak of Fab_2_ and Fab_2_-comprising C_H_2 domains. The exact nature of these species could not be determined due to the high amino acid sequence variability of their variable portions and was derived from the identified cleavage sites. (**C**) SpeBm cleavage sites are mapped to a schematic of an IgG1 antibody for all four subclasses in a combined manner. Whether the cleavage also occurs under physiological conditions is specified in the respective small box with a red cross. (**D**) Likelihood of cleavage sites calculated for the hinge region (upper sequence) and the joint region (lower sequence) of IgG1. High green values suggest likely cleavage, whereas low red values suggest unlikely cleavage. Values were calculated based on amino acid occurrences relative to natural abundance of combined tryptic and GluC PICS data, considering P3 to P3′. Black vertical lines show experimentally observed cleavages.

As suggested by N-terminomics, the IgG heavy chain (IGHG) appears to be readily cleaved by SpeBm. This was not expected, because although IgG has been proposed as a substrate for SpeB in earlier reports, a recent study found that IgGs are probably not in vivo substrates [22]. This report claims that SpeB proteolysis requires prior reduction of intermolecular disulfides in IgG and is thus resistant to SpeB in its native state. Prompted by these controversies, our proteomics findings, and the fact that IgG may play an important role in streptococcal infection, we set out to assess SpeBm-induced cleavage in preparations of purified human IgG of all subclasses. To this end, we incubated IgG with activated SpeBm under physiological and reducing conditions and analyzed the generated protein species, especially focusing on the Fc-region due to its non-variable amino acid sequence. More precisely, proteolytically processed IgG fragments were separated by means of reversed-phase chromatography and subjected to high-resolution mass spectrometry. In this way, we addressed the cleavage of IgG under both conditions at a time point of three hours. Upon the addition of reducing agents, we were able to detect a number of specific cleavage sites in IgG1, IgG2, IgG3, and IgG4 (Figure 5). The most abundant species in all subclasses resulted from a cleavage in the upper hinge region, more specifically at a XXXX|CPXX motive just N-terminal of an intermolecular disulfide bond. This specific cleavage led to the generation of Fc/2 subunits from all IgG subclasses (Figure 5A). In addition, IgG1 and IgG3 were susceptible to an additional cleavage C-terminal to the intermolecular disulfide bonds in the hinge region at a PAPE|LLGG motive. Interestingly, we could detect another readily cleaved position in the linking region of the globular C_H_2 and C_H_3 domains in all four subclasses leading to the generation of Fc/4 subunits of approximately 12.5 kDa in size. This was accompanied by a second frequently cleaved site in the upper region of this linking region. To simulate a more realistic in vivo scenario, we investigated proteolysis of IgG subclasses by activated SpeBm under physiological conditions at neutral pH and providing no reducing agents. Surprisingly, IgGs were still degraded efficiently by SpeBm (Figure 5B). However, solely the two cleavage sites in a likely unstructured joint region of the C_H_2 and C_H_3 domain were readily accessible and cleaved by the protease, which led to the release of Fc/4 variants identical to the products identified under reducing conditions. Of note, one of these two cleavage sites (KTIS|KTKG) was also identified in the N-terminomics of plasma samples. The most prominent cleavage site in the upper hinge region upon addition of a reducing agent (XXXX|CPXX) was not found to be cleaved under physiological conditions. However, cleavage at the PAPE|LLGG motive in the hinge creating Fc/2 fragments could be identified in low quantities compared to the amounts detected after proteolysis in a reducing environment. A schematic overview of SpeBm cleavage sites in human IgG subclasses under reducing and physiological conditions is illustrated in Figure 5C. A list of all cleavage sites and the corresponding masses and mass errors in the conducted experiments is provided in Table S1. To see whether the cleavage of SpeB in IgGs is mainly governed by the accessibility of these sites or SpeB’s amino acid specificity, a probability score was calculated based on the acquired PICS data (Figure 5D). Considering P3 to P3′, we aimed to illustrate the likelihood of cleavages in the hinge and the joint region of IgG1. Indeed, the suggested cleavage sites (green color; Figure 5D) clearly matched the sites found to be cleaved on the basis of our experimental data.

## 3. Discussion

In this study, we assessed the proteolytic properties and identified the protein substrates of the cysteine protease SpeB, a major virulence factor of group A streptococcus (GAS). Although substantial efforts have been undertaken over the last several decades to study this key protease, we revisited this topic employing a number of complementary state-of-the-art HPLC-MS workflows to provide a comprehensive and unbiased characterization. 

Initially, we conceived a protocol to efficiently express and purify SpeBz in a recombinant manner. After purification, SpeBz was immediately inhibited by the addition of MMTS and subjected to SDS-PAGE and HPLC-MS analysis (Figure 1A,B and Figure S1A,B), confirming that the majority of SpeB was still in its zymogen form. However, a minor percentage of purified SpeB was already cleaved within its pro-domain at a site distinct from the mature N-terminus (Figure 1C), indicating iterative cleavage during autoactivation. This site was already suggested by Doran et al. to be autocatalytically processed [7]. Moreover, SpeBz was found to be of excellent purity, as no major protein contaminants were identified in either experiment. SpeBz could be efficiently transformed into its mature form, SpeBm, within 30 min at 37 °C, as verified by SDS-PAGE and HPLC-MS analysis (Figure 1 and Figure S1B). Interestingly, we detected minor amounts of dimerized SpeBm in the HPLC-MS analysis. Although SpeBm is known to be dimeric [10], the stability of this noncovalent interaction under such harsh conditions (approx. 40% ACN and 0.05% TFA at 70 °C) is unlikely. Presumably, this protein species is caused by a covalent linkage of two SpeBm molecules via their active site cysteines. Additionally, the observed average mass would indicate this loss of two hydrogen atoms by a mass shift of 2.0 Da. Consistent with this interpretation, dimerization was solely detected in measurements of SpeBm and not in those of SpeBz, where the access to the active site cysteine is hindered.

After confirming purity and determining specific activity, the sequence specificity profile of SpeBm was comprehensively assessed by proteomic specificity profiling, namely the PICS method. Based on published protocols [31,32], we validated the establishment in our lab thoroughly after study-specific optimization of the workflow (Figure S2). Thereafter, SpeBm was incubated with peptide libraries created by trypsin and GluC digestions of the entire *E. coli* proteome. Evaluation of these data revealed a well-defined sequence specificity profile of SpeBm with a clear overlap of both independently prepared libraries. Using almost 2000 identified peptide substrates, we were able to create heatmaps and sequence logos to visualize the SpeBm sequence specificity in great detail (Figure 2). One of the most prominent features is the preference for substrates with a hydrophobic amino acid residue at the P2 site. This was already proposed by early studies examining the autocatalytic cleavage sites in the pro-domain of SpeBz that carry isoleucine or valine at four of the five sites reported at that time [7,12]. An additional four autocatalytic cleavage sites identified later by Chen et al. [11] strengthened this hypothesis, even if methionine and tyrosine were also identified at the P2 site. In our study, we performed the first unbiased approach to portray the specificity of SpeBm, studying thousands of potential peptide substrates and neglecting previously characterized autocatalytic cleavage sites. Indeed, we could substantiate the preference for hydrophobic residues at the P2 site in both PICS experiments (Figure 2). The highest enrichment was identified for isoleucine and valine, which is in agreement with findings derived from the sites of autocatalysis. Despite its comparable hydrophobicity, leucine was not preferred at P2 but was accepted. This finding is in line with a previous report that observed drastic differences in the k_cat_/K_m_ values for either leucine or isoleucine at the P2 site [13]. In addition, we could observe a pronounced specificity for phenylalanine and tyrosine residues at P2. Potential aromatic interactions of substrate and protease would be in good agreement with a proposed binding pocket for the P2 residue comprising one tryptophan and two phenylalanine residues [41]. Besides, our data suggest that methionine also possesses sufficient hydrophobicity to be efficiently recognized by the S2 site of SpeBm (Figure 2A,C and Figure 3A,C). Due to the nature of the initially reported sites of autocatalysis and the data reported by Nomizu et al. [13], a preference for positively charged residues at the P1 site was proposed. Although we could identify the efficient degradation of peptides comprising a lysine at P1 (Figure 2B,D), this feature seemed to be less specific, as a variety of hydrophilic, negatively charged, and even moderately hydrophobic residues, i.e., alanine, were enriched at this position. Intriguingly, in both PICS libraries, an interesting picture of the prime sites from P4′ to P6′ emerged. Specifically, negatively charged residues, namely aspartic and glutamic acid, were highly enriched, which suggests a further recognition mechanism of peptide substrates by S4′ to S6′ sites in SpeBm. Furthermore, prolines were specifically enriched in the prime positions from P4′ to P6′, wherein the P4′ position showed the most pronounced enrichment (Figure 2). On the contrary, proline was completely absent at the P1 position, and only one proline was determined in the P1′ site in nearly 2000 identified peptide substrates (Figure 2, Tables S3 and S4). Thus, proline in either one of the two positions next to the scissile bond completely abrogated proteolysis. The previous finding that proline at the P3 position leads to a 180-fold decrease in activity, reported by Nomizu et al. [13], could not be corroborated using our PICS approach. In fact, the prime sites from P6 to P3 showed little to no specificity for certain amino acids in either peptide library examined.

The discrepancy in the number of cleaved substrates identified between the two libraries may be explained by the more pronounced digestion of peptide substrates derived from the GluC library, considering the finding that SpeB prefers negatively charged residues at P3´to P6´. Therefore, some product species originating from SpeB digestion may not be experimentally accessible, as these species are too small for HPLC-MS analysis both in terms of retention on the reversed phase column and in terms of their *m*/*z* being too small for MS detection.

To get an insight into the cleavage of relevant proteins in their native state, we conducted an approach mostly relying on the positional proteomics method HUNTER to investigate monocytic secretome and plasma samples. Indeed, we could substantiate the specificity profiles obtained by our preceding peptide-based analyses in both biological samples (Figure 3 and Figure 4). The slight discrepancy in the overall agreement of the specificity profiles may be due to the relatively lower number of cleavage events identified compared to peptidic substrate libraries. What still stands out in the comparison of the specificity profiles is the enrichment in cysteine residues at the C-terminal part of the scissile bond in the N-terminomics data (Figure 4). Since most of the cysteines in proteins are not thought to be engaged in disulfide bonds [42,43], this may potentially indicate a slight preference for non-disulfide-linked cysteine residues. However, since we did not detect any of this cysteine specificity in our PICS data, this may not indicate an actual preference for cysteine residues but may arise from an advancing digestion until a disulfide bond, and the fact that for subsequent evaluation, only the C-terminal portion of the cleavage site could be experimentally assessed.

Beyond strengthening our understanding of SpeB’s amino acid specificity, N-terminomics experiments were targeted to identify native SpeBm substrates in proteins present in the monocytic secretome. On the one hand, this specific sample was chosen due to the availability of a suitable cell line, i.e., MOLM-13, which resembles the monocytic phenotype and can be propagated to numbers sufficiently high to obtain appropriate starting material. On the other hand, these cells may secrete similar proteins to those released from cells of the innate immune system and thus may have potential implications during streptococcal infection. In total, N-terminomics of these samples revealed 262 unique cleavage events in 180 individual proteins, as listed in Table S6. As a first step, these proteins were ranked by the number of individual cleavage events identified. Surprisingly, among two other proteins, cystatin C showed the highest number of cleavage sites. Given its low molecular weight of approx. 13 kDa, this is even more astonishing. Cystatin C is an extracellular cysteine protease inhibitor with antimicrobial properties. Interestingly, Björck et al. show that a peptide derivative mimicking cystatin C reportedly inhibited the growth of GAS, among other bacteria [44]. Thus, efficient clearance of this protein during infection may be favorable for the activity of bacterial proteases and the unhindered growth of GAS. Indeed, four out of five identified cleavage sites (UniProt Accession: P01034) in this study were within the N-terminal inhibiting region [45], sequentially degrading the L_35_VG motive that was also the basis of the antimicrobial peptide developed in the above-mentioned study of Björck et al. [44]. The remaining cleavage site was identified within another region responsible for inhibiting papain-like cysteine proteases [45], specifically at the IV_83_|AG sequence motive. Of note, the activity of IdeS, another major cysteine protease of GAS, is reportedly increased in the presence of cystatin C, for which it acts as an activating cofactor [46]. This could suggest a sequential action of these two immunoglobulin-degrading proteinases; in the early phase of infection, cystatin C-stabilized IdeS may dominate GAS’s protease activity, followed by SpeB expression and cystatin C degradation at a later stage of infection.

Other multiply cleaved proteins obtained from secretome were QSOX1, a secreted cysteine oxidase involved in shaping the extracellular matrix (ECM) [47], and PPIA, a multifunctional peptidyl-prolyl isomerase with implications in multiple signaling events, including inflammation and chemotaxis [48]. To get a deeper insight into the residual 177 cleaved proteins, we additionally performed enrichment analysis of the cleaved proteins versus the identified protein background based on their reported molecular function (Figure 3D). Besides a high enrichment for secreted proteins, we found that a number of proteins assigned to two clusters containing various signaling proteins were enriched. In addition, multiple proteins of the redox system, including those containing thioredoxin domains, were efficiently degraded. This enrichment analysis and the surprisingly high number of protein targets strengthen the evidence that SpeB may be able to perturb host cells and their ECM in a more diverse manner beyond the proteolytic degradation of anti-microbial proteins such as IgGs, as suggested earlier [18,21].

N-terminomics of plasma could further expand our insights into SpeB’s host substrates. In essence, 135 unique cleavage events were assigned to 53 proteins. By ranking these proteins according to the number of cleavage sites, we reported an astonishing 18 cleavage sites in albumin (Figure 4C). This is somewhat counterintuitive, as the usually high abundance of albumin may affect the efficient cleavage of other, perhaps more relevant substrates. However, one may speculate that some albumin molecules in plasma are present as already damaged or partially unfolded forms and thus are rendered amenable to SpeB proteolysis. Given the high quantities of albumin, these cleavage sites may still be identified by our N-terminomics experiments. Additionally, proteins of the complement system, e.g., C3 and C4, showed multiple cleavage sites. As proposed by Rasmussen et al., complement activation during streptococcal infection is thought to be downregulated by the streptococcal M protein that is binding to factor H and the C4b-binding protein, especially during an early phase of infection [9]. In addition, during this time of infection, bacterial proteins GRAB (protein G-related alpha 2M-binding protein) and M-like proteins were shown to recruit protease inhibitors A2MG and kininogens to the cellular surface, which may lead to a generally downregulated proteolytic activity at the streptococcal surface. During a later stage of infection in which SpeB expression is induced, the reported cleavage of proteins of the complement system by SpeB may be an additional mechanism of GAS to evade complement activation. One of these mechanisms was proposed by Terao et al., who were able to show that SpeB rapidly degrades C3b and this is of importance for pathologies caused by GAS [49]. As proteolysis of proteins of the complement system may have opposing effects depending on the actual site, we looked into the respective cleavage sites of SpeB in the C3 protein (UniProt Accession: P01024) identified in this study; two cleavage sites within the C3 beta chain (VF_375_|VT and YS_447_|TV) and several cleavages throughout the C3 alpha´ chain may suggest inhibition of the active C3b proteoform. However, we also detected a number of cleavage events that are in the vicinity of the activating site cleaved by C3 convertase within the C3 alpha chain, presumably releasing active C3a anaphylatoxin. A similar picture could be observed for C4: we detected both activating-like cleavages of C4 creating C4a anaphylatoxin and C4b. However, several additional cleavages in the region of C4b could be detected in the C4 alpha, beta, and gamma chains, suggesting an overall inhibitory effect on C4b.

In agreement with the literature, we could also identify A2MG and kininogen 1 to be cleaved by SpeB [50,51]. However, in A2MG, we identified cleavage sites distinct from its bait region as identified earlier [51]. This potential clearance of antimicrobial proteins by SpeB proteolysis was also reflected by the great proportion of cleaved proteins involved in defense and immunity (Figure 4D). Unfortunately, the exact role of the cleaved proteins identified by our means still remains elusive, as it was not within the scopes of this study. However, we provide an extensive library of potential protein substrates present in relevant biological surroundings and provide the exactly mapped cleavage sites in order to prompt and facilitate further research. Moreover, we could corroborate earlier findings that vitronectin, fibronectin [52], fibrinogen [53], and plasmin [54] are degraded by the action of SpeBm. In addition, our data confirm that IgA, IgM, and IgG are likely in vivo substrates under physiological conditions [18] and determine the actual cleavage sites within these proteins. Additionally, SpeBm cleavage was examined in greater detail for human polyclonal IgG subclasses, as its implications in *S. pyogenes* infections are controversially discussed. While some reports determined efficient cleavage of IgG by SpeB and therefore proposed this as a putative immune evasion mechanism of the pathogen, they may not have fully considered the influence of the reducing environment in which their experiments were performed [17,21]. A more recent report by Persson et al. opposes these findings and claims that human IgGs are likely no proteolytic target of SpeBm in an in vivo scenario or under physiological conditions [22]. In our study, we confidently detected several cleavage sites in purified IgG incubated with SpeBm under reducing conditions (Figure 2, Table S1). Astoundingly, the anticipated cleavage site in IgG (ELLG|GPSV) that was reported earlier [18,19] could not be detected in any subclass; however, this is in line with leucine not being favored in P2, the major specificity determinant. In contrast, a prominent cleavage site in the hinge region was defined at an XXXX|CPXX motive of each subclass examined. Potentially, the reported ELLG|GPSV cleavage site could be an experimental error created by contaminations in SpeB preparations with the IdeS protease, which reportedly cleaves IgG uniquely at this position [55]. The main SpeB cleavage site determined in this study (XXXX|CPXX) is distinct but in close proximity to the one of papain [56]. Considering the similar specificity for hydrophobic residues at the P2 site of both papain and SpeB, the difference in cleavage sites was remarkable and points towards distinct specificity profiles. However, the second minor cleavage site at a conserved PAPE|LLGG motive that was identified in this study (Figure 2) is identical to a site reported for the papain protease upon extended incubation [56]. Intriguingly, even under physiological conditions providing no reducing agents, SpeBm showed efficient degradation of IgG, clipping off the C-terminal C_H_3 domain of all subclasses. Indeed, these identified cleavage sites (IS|KX and IE|KT) are also in clear agreement with our specificity profiling data and the likelihood for cleavage as calculated in Figure 5D. Therefore, IgG subclasses may still represent a target for SpeBm in an ongoing infection to evade the host´s immune response, yet the prominent cleavage within the hinge detected in our assays featuring a reducing environment was not accessible by SpeBm under non-reducing conditions. Clearly, further research needs to be conducted to draw detailed conclusions on the biological relevance of these findings. From an analytical point of view, SpeBm efficiently generates Fc/4 and Fc/2 subunits that are readily separated by reversed-phase HPLC and may be examined at high resolution due to their low mass of approximately 12.5 and 25 kDa, respectively. This may also find application in the analysis of biopharmaceuticals, e.g., monoclonal antibodies and polyclonal IgGs as a so-called middle-up/down protease, which aids the characterization of these large biomolecules [24,36,57]. 

In conclusion, we conceived an efficient and straightforward protocol to obtain SpeBz and SpeBm of excellent activity and purity. Relying on advancements in protease characterization based on HPLC-MS methods, we were able to portray SpeB’s amino acid specificity, showing a prominent enrichment of hydrophobic and aromatic residues at its P2 site, preference for negatively charged amino acids at the P3′ to P6′ sites, and efficient inhibition of cleavage by prolines on either side of the scissile bond. N-terminomics of secretome and plasma samples revealed more than 200 human host proteins being substrate to SpeB in their native state, including several proteins of the ECM and the complement system, protease inhibitors, immunoglobulins, and proteins linked to inflammation. While this study does not aim to unravel the role of individual proteins during GAS infection, we provide an exploratory view on SpeB substrates with potential relevance for streptococcal virulence, and we could profile SpeB’s proteolytic properties in an unprecedented manner.

## 4. Materials and Methods

### 4.1. Recombinant Expression and Purification of SpeBz

SpeBz was expressed, purified, and activated similarly to published reports [38,39]. However, the protocol was carefully optimized to increase yield and specific proteolytic activity, as described below.

#### 4.1.1. Cloning and Expression

The coding sequence for SpeBz (UniProt entry: P0C0J0) was taken from the ENA database (Accession: M86905) [58,59], the signal peptide was removed, and the sequence was synthesized by Eurofins Genomics (Ebersberg, Germany). The amino acid sequence is available in Figure S1. This sequence was cloned into a pET22b plasmid employing restriction digestion by NdeI and XhoI and subsequent T4 DNA ligase ligation (all from Thermo Fisher Scientific, Vienna, Austria), adding a C-terminal His6-tag to the SpeBz amino acid sequence. The correct insertion and orientation of the coding sequence were confirmed by sequencing (Eurofins Genomics). Rosetta 2 (DE3; Merck Millipore, Darmstadt, Germany) *E. coli* was transformed and grown as a polyclonal culture in 4 mL standard LB medium (Roth, Karlsruhe, Germany) supplemented with 100 µg·mL^−1^ ampicillin and 20 µg·mL^−1^ chloramphenicol (LB–Amp–C) at 37 °C (both antibiotics were purchased from AppliChem, Darmstadt, Germany). This starting culture was grown overnight, and 250 µL was used to inoculate 50 mL LB–Amp–C medium, which was incubated at 37 °C to an OD_600 nm_ of 0.8–1.0. Eventually, SpeBz expression was induced by addition of 1.0 mmol·L^−1^ IPTG (ForMedium, Norfolk, UK) and incubation at 25 °C for 5 h under continuous shaking. Cells were harvested by centrifugation at a relative centrifugal force of 17,500× *g* for 10 min at 4 °C. The pellet was stored at −20 °C.

#### 4.1.2. Purification and Activation

0.20 g of the frozen pellet was dissolved in 1.0 mL ice-cold wash buffer (100 mmol·L^−1^ TRIS HCl (pH 8.0; Serva, Heidelberg, Germany), 100 mmol·L^−1^ NaCl (Sigma-Aldrich)). An amount of 10 mmol·L^−1^ MgSO_4_ (Merck Millipore) and a spatula tip of DNase I (AppliChem) were added, and the mixture was sonicated three times for 45 s at 40% power and 50% interval (SONOPULS, Bandelin, Berlin, Germany). After sonication, the cell suspension was centrifuged at 16,000× *g* for 20 min at 4 °C, and the supernatant was transferred onto 200 µL Ni-NTA (Qiagen, Hilden, Germany) and incubated for 1 h at 4.0 °C. The column was washed two times with 500 µL wash buffer, and SpeBz was eluted four times with 200 µL elution buffer (50 mmol·L^−1^ NaOAc, 100 mM NaCl, pH 4.5; both from Sigma-Aldrich, Vienna, Austria). Concentration was determined at a wavelength of 280 nm on a NanoDrop 2000 UV/Vis spectrophotometer (Thermo Fisher Scientific) and diluted to 1.0 mg·mL^−1^ in elution buffer. The expression and purification of SpeBz were illustrated by SDS-PAGE analysis (Figure S1A). To determine the properties of SpeBz, proteolytic activity was inhibited immediately after purification by addition of 5.0 mmol·L^−1^ methyl methanethiosulfonate (MMTS; Thermo Fisher Scientific). If not stated otherwise, SpeBz was activated without prior addition of MMTS by incubation at 37 °C for 30 min under gentle shaking and was used for all following experiments. SpeBz was determined as >95% pure on an SDS-PAGE stained with Coomassie Brilliant Blue G250 (AppliChem), as illustrated in Figure S1B. SpeB aliquots were frozen in liquid nitrogen and stored at −20 °C until further use.

#### 4.1.3. Activity Test

Proteolytic activity of SpeBm was tested by an azocasein assay [60]. Corresponding data are listed in Table S2 and illustrated in Figure S1C. Briefly, activity was determined by an increase in the absorbance at 366 nm of released azo-dye from proteolytically cleaved azocasein over time. An amount of 20 µL of 0.25 mg·mL^−1^ SpeBm was added to 160 µL of 3.0 mg·mL^−1^ azocasein (Sigma-Aldrich) in PBS buffer (Merck Millipore) supplemented with 5.0 mmol·L^−1^ dithiothreitol (DTT; Sigma-Aldrich) and 5.0 mmol·L^−1^ ethylenediaminetetraacetic acid (EDTA; Merck Millipore) and incubated at 37 °C. Samples were taken between 15 s and 8 min and quenched with 40 µL of ice-cold 0.50 mol·L^−1^ trichloroacetic acid (Sigma-Aldrich). The absorbance at 366 nm was measured using a NanoDrop2000 UV/Vis spectrophotometer. A standard curve was obtained by total digestion of azocasein by SpeBm and employed to determine the specific activity of SpeBm.

### 4.2. PICS Analysis

#### 4.2.1. Library Preparation

Proteome-derived peptide libraries were prepared as described previously in detail [32,61]. Briefly, *E. coli* protein lysate was obtained by solubilizing a pellet of bacteria in 6.0 mL 10-mmol·L^−1^ HEPES buffer (pH 7.1; Sigma-Aldrich), including cOmplete protease inhibitor cocktail without EDTA (Roche, Basel, Switzerland). Cells were lysed by ultrasonication for 5 min at a duty cycle of 70% (Sonifier S-250A, Branson Ultrasonics, Brookfield, CT, USA). Cell debris was removed by centrifugation at 20,000× *g* for 20 min. The supernatant was collected, and proteins were precipitated by addition of a 7-fold excess of ice-cold acetone (Merck Millipore). Samples were mixed thoroughly and incubated at −20 °C for 3 h. Subsequently, the precipitate was pelleted by centrifugation at 4000× *g*, and the supernatant was removed. The pellet was reconstituted in 50 mmol·L^−1^ TEAB (triethylammonium bicarbonate, pH 8.5, Sigma-Aldrich) to a concentration of 5.0 mg·mL^−1^. Before digestion, proteins were denatured by addition of TCEP (tris(2-carboxyethyl)phosphine; Sigma-Aldrich) to a final concentration of 5.0 mmol·L^−1^ and incubated at 60 °C for 30 min, shaking horizontally at 900 rpm on a thermoshaker (Thermomixer comfort, Eppendorf, Germany). After denaturation of proteins, digestion with library proteases trypsin and GluC, both from Promega (Madison, WI, USA), was performed at an enzyme-to-substrate ratio of 1:50 (*w*/*w*) for 18 h at 37 °C while shaking at 850 rpm.

#### 4.2.2. SpeB Cleavage of Peptide Substrates

For SpeB proteolysis, 100 µg bacterial library peptides was diluted in 175 mmol·L^−1^ ammonium acetate (pH 6.9; VWR chemicals, Radnor, PA, USA) to a concentration of 1.0 mg·mL^−1^ and supplemented with 5.0 mmol·L^−1^ TCEP. Digestion with SpeB was performed at an enzyme-to-substrate ratio of 1 U·µg^−1^ for 3 h at 37 °C while shaking at 850 rpm on a thermoshaker. PICS experiments were carried out with the commercially available SpeBm enzyme (FABulous; Genovis, Lund, Sweden). Eventually, the reaction was quenched by alkylation of cysteines using iodoacetamide (IAA; Sigma-Aldrich) at a final concentration of 50 mmol·L^−1^ and incubation in the dark for 30 min at 22 °C. Digested and alkylated samples were desalted using 100 µL Pierce C18 tips (Thermo Fisher Scientific) according to the manufacturer’s instructions. Eluted peptides were dried using a vacuum centrifuge at 30 °C and subsequently resuspended in 100 mmol·L^−1^ TEAB (pH 8.5) to a concentration of 1.00 mg·mL^−1^. 

#### 4.2.3. Dimethyl Labeling

Labeling of peptides was based on dimethylation of primary amines as follows: light (control; Sigma-Aldrich) and heavy formaldehyde (C^13^D_2_O for SpeBm; Sigma-Aldrich), respectively, was added to a sample to a concentration of 40 mmol·L^−1^ supplemented with 20 mmol·L^−1^ sodium cyanoborohydride (Sigma-Aldrich). The samples were incubated at 22 °C while shaking at 850 rpm on a thermoshaker for 1 h. Subsequently, the procedure was repeated to reach a final concentration of 80 mmol·L^−1^ formaldehyde supplemented with 40 mmol·L^−1^ sodium cyanoborohydride. The samples were incubated at 22 °C and 850 rpm on a thermoshaker for an additional 1 h. The reaction was quenched by adding TRIS HCl (pH 7.5) to a concentration of 100 mmol·L^−1^, followed by incubation for 15 min. Labeled samples were pooled and desalted using 100 µL Pierce C18 tips according to the manufacturer’s instructions. Eluted peptides were dried at 30 °C using a vacuum centrifuge. Based on the amount of starting material, samples were resuspended in H_2_O + 0.10% formic acid (FA; Sigma-Aldrich) to a concentration of 4.00 mg·mL^−1^ and subjected to HPLC-MS analysis. 

### 4.3. N-Terminomics

#### 4.3.1. Intact Protein Library Preparation

N-terminomics experiments were conducted highly similarly to previously published protocols [34,62]. The basis for the conducted protocol is available online: http://clip2.sites.olt.ubc.ca/files/2016/05/16-05-Overall-Lab-TAILS-Protocol-v4.pdf (accessed on 1 June 2021). Briefly, MOLM-13 cells were grown in four T175 flasks to a confluence of about 85% and subsequently washed three times with PBS (37 °C) and incubated for 2 h in serum-free medium (37 °C). This medium was discarded and replaced by fresh serum-free medium, phenol red-free, and incubated overnight. The conditioned medium was harvested, immediately adding Roche complete protease inhibitor cocktail including 10 mmol·L^−1^ EDTA, and put on ice. The collected supernatant was filtered using 0.2 µm pore size to remove any particulate matter. Subsequently, proteins were concentrated by ultrafiltration at 4 °C using 3 kDa cut-off filters (Amicon, Ultra-15; Merck Millipore). Proteins retained within the 3 kDa cut-off filters were washed three times with 10 mL of 100 mmol·L^−1^ HEPES (pH 7.5).

Plasma was obtained from anonymous blood samples discarded after plasmapheresis. Importantly, in the case of anonymous blood donations, Austrian national regulations do not require informed consent or approval by the local ethics committee. Plasma samples obtained from two individual donors were depleted of primary amine-containing small molecules by buffer exchange on 3 kDa cut-off filters to 100 mmol·L^−1^ HEPES (pH 7.5). Nucleic acids were digested by addition of benzonase (Merck Millipore) and subsequent purification of proteins by ultrafiltration on 3 kDa cut-off filters. Eventually, protein content of both plasma and secretome samples was determined by a BCA assay (Thermo Fisher Scientific).

#### 4.3.2. Digestion of Intact Proteins and Isotope Labeling

600 µg of monocytic secretome and 2 mg of purified plasma (reconstituted in HEPES, pH 7.5 as detailed above) were equally split into two reaction tubes, one serving as control whereas the other was supplemented with SpeBm at an enzyme-to-substrate ratio of 1:50 (*w*/*w*) (0.25 U·µg^−1^), and incubated for 16 h at 37 °C. All following steps were performed equally for both the control and the SpeBm treated sample. Proteolysis was quenched by alkylation of the active cysteine of SpeB by addition of IAA to a concentration of 5.0 mmol·L^−1^ in both tubes and incubation at 21 °C for 30 min. Subsequently, to denature all proteins, guanidine HCl (Thermo Fisher Scientific) was added to a final concentration of 3.5 mol·L^−1^ and DTT to a concentration of 10 mmol·L^−1^. After the pH was confirmed to be approx. 7.5, the reaction was incubated at 37 °C for 60 min, followed by addition of IAA to a concentration of 30 mmol·L^−1^ and incubation at room temperature in the dark for another 30 min. The reaction was quenched upon the addition of DTT to 30 mmol·L^−1^ followed by 20 min incubation at 21 °C. Next, primary amines were labeled by reductive dimethylation, as described in the previous section, with slight adjustments to ensure complete labeling of intact proteins instead of peptides. The initial incubation step was performed overnight, and the additional incubation step was conducted for 2 h. Reactions were quenched by supplementing the reactions with 100 mmol·L^−1^ TRIS HCl (pH 7.5) followed by 1 h incubation at 37 °C. Light- and heavy-labeled samples were mixed and precipitated by addition of chloroform/methanol, followed by extensive washing with cold methanol. The pellet was resuspended in 100 mmol·L^−1^ HEPES (pH 7.5) and split into two aliquots: one aliquot was digested with trypsin at a substrate-to-protein ratio of 1:25, and the other aliquot with GluC at a ratio of 1:20. Both reactions were incubated overnight at 37 °C.

#### 4.3.3. Enrichment of Novel N-Termini

Enrichment of novel N-termini generated by SpeBm was conducted according to a recently published protocol (HUNTER) by Weng et al. [34]. Briefly, samples were adjusted to 40% ethanol (Merck Millipore) followed by addition of undecanal (Alfa Aesar, Thermo Fisher Scientific) at a peptide-to-undecanal ratio of 1:30 (*w*/*w*). The reaction was supplemented by sodium cyanoborohydride (30 mmol·L^−1^) and incubated for 2 h at 37 °C. The solution was acidified by addition of 0.5% trifluoroacetic acid (TFA; Sigma-Aldrich) in 40% ethanol to a pH of approx. 3.5. Undecanal-tagged N-termini and excess of undecanal were removed by C18 solid-phase extraction (SUPRA-CLEAN C18-S 500 mg; PerkinElmer, Waltham, MA, USA). The flow-through was collected, dried by vacuum centrifugation, resuspended in H_2_O + 0.1% TFA, and desalted using 100 µL Pierce C18 tips according to the manufacturer’s instructions.

### 4.4. Cleavage of Human IgG subclasses

As previously described [24], polyclonal IgGs were purified using protein G from plasma samples that were obtained as stated in the above paragraphs. For SpeB proteolysis, polyclonal IgGs were diluted in 150 mmol·L^−1^ ammonium acetate (pH 6.9) to a concentration of 1.0 mg·mL^−1^. Samples featuring reducing conditions were supplemented with 5 mmol·L^−1^ TCEP. Digestion with SpeB was performed at an enzyme-to-substrate ratio of 1:50 (*w*/*w*) (0.25 U·µg^−1^) for 3 h at 37 °C while horizontally shaking at 850 rpm on a thermoshaker. Samples were quenched by addition of IAA to a concentration of 10 mmol·L^−1^ and incubation at 21 °C in the dark for 30 min. Samples were subjected to HPLC-MS analysis without prior purification.

### 4.5. High-Performance Liquid Chromatography

Chromatographic separation of SpeBz and SpeBm, as well as digested human IgGs, was carried out on a capillary HPLC instrument (UltiMate™ U3000 RSLC, Thermo Fisher Scientific, Germering, Germany) employing a reversed-phase column (BioResolve polyphenyl, 150 × 2.1 mm i.d., 2.7 μm particle size, solid core, 450 Å pore size, Waters, Milford, MA, USA). A flow rate of 200 µL∙min^−1^ and a column oven temperature of 70 °C were used. An amount of 10.0 µL of SpeBz or SpeBm (0.1 mg∙mL^−1^) was injected using in-line split-loop mode. A multi-step linear gradient of mobile phase solutions A (H_2_O (obtained from a Milli Q Integral 3 system; Merck Millipore) + 0.050% TFA) and B (acetonitrile (ACN; HiPerSolv, VWR Chemicals) + 0.050% TFA) was applied: 20.0–28.0% B in 5 min, 28.0–50.0% B in 35 min, 80.0% B for 5 min, and 20.0% B for 15 min. For digested IgGs, 10.0 µL of sample (1.0 mg∙mL^−1^) was injected using in-line split-loop mode. IgG fragments were separated as follows: 5.0% B for 5 min, 5.0–29.0% B in 5 min, 29.0–32.0% B in 15 min, 32.0–80.0% B in 15 min, 80.0% B for 5 min, and 5.0% B for 15 min. UV detection was carried out at 214 nm using an 11 µL flow cell.

For chromatographic separation of peptides generated by PICS and the N-terminomics workflow, a nanoHPLC instrument (UltiMate™ U3000 RSLCnano, Thermo Fisher Scientific, Germering, Germany) was employed. The flow rate of the nano-pump was set to 300 nL∙min^−1^ and the column oven temperature to 50 °C. Separation of PICS samples was performed on an Acclaim™ PepMap™ 100 C18 column (500 mm × 75 µm i.d., 3.0 μm particle size, Thermo Fisher Scientific, Sunnyvale, CA, USA). An amount of 1.0 µL of sample was injected using full-loop mode. A multi-step linear gradient of mobile phase solutions A (H_2_O + 0.10% FA) and B (ACN + 0.10% FA) was applied as follows: 1.0–22.0% B in 200 min, 22.0–40.0% B in 40 min, 80.0% B for 20 min, and 1.0% B for 40 min. Separation of N-terminomics samples was conducted on a 2000 mm μPAC™ C18 column preceded by a μPAC™ trap column (both from PharmaFluidics, Ghent, Belgium). An amount of 1.0 µL of sample was injected using µL pick-up mode on a 3.0 µL loop. A multi-step linear gradient of mobile phase solutions A (H_2_O + 0.10% FA) and B (ACN + 0.10% FA) was applied by the nano-pump as follows: 1.0% B for 10 min, 1.0–3.0% B in 5 min, 3.0–21.0% B in 175 min, 21.0–50.0% B in 35 min, 80.0% B for 15 min, and 1.0% B for 60 min. The loading pump delivered a constant isocratic flow of 4.0 µL∙min^−1^ of 1.0% ACN + 0.10% TFA. A ten-port switching valve within the column oven was used to switch at time points 10 min and 290 min, and mass spectrometric data were recorded in between these time points.

### 4.6. Mass Spectrometry

Recombinant SpeBz, SpeBm, and digested human IgG subclasses were analyzed on a Thermo Scientific™ QExactive™ benchtop quadrupole-Orbitrap^®^ mass spectrometer equipped with an Ion Max™ source with a heated electrospray ionization (HESI) probe (Thermo Fisher Scientific, Bremen, Germany), as well as an MXT715-000—MX Series II Switching Valve (IDEX Health & Science, LLC, Oak Harbor, WA, USA). Mass spectrometric data of IgG subunits were acquired with instrument parameters described earlier [24]. For SpeBz and SpeBm data acquisition, some parameters were adjusted; the resolution was set to 17,500 at *m*/*z* 200 in a mass range of *m*/*z* 800 to 3000, and the source heater temperature was lowered to 120 °C.

HPLC-MS measurements for PICS and N-terminomics were conducted on a quadrupole-Orbitrap^®^ hybrid mass spectrometer (Thermo Scientific™ QExactive™ Plus benchtop quadrupole-Orbitrap^®^ mass spectrometer) equipped with a Nanospray Flex™ ion source both from Thermo Fisher Scientific (Bremen, Germany) and a SilicaTip™ emitter with 360 µm o.d., 20 µm i.d., and a tip i.d. of 10 µm (New Objective, Woburn, MA, USA). PICS data were acquired with instrument settings stated in Eckhard et al. [63]. N-terminomics data acquisition was optimized by increasing the MS^1^ injection time to 120 ms, the MS^2^ target to 2^5^, and the MS^2^ maximum injection time to 150 ms. Both instruments were mass calibrated using Pierce™ LTQ Velos ESI Positive Ion Calibration Solution from Life Technologies (Vienna, Austria) and AHFP (ammonium hexafluoride, Sigma-Aldrich) for higher mass ranges.

### 4.7. Data Analysis

MS/MS data of PICS and N-terminomics approaches were evaluated using MaxQuant 1.6.12.0 [64]. Raw files were individually analyzed using adjusted dimethyl labeling ($D_{4}C_{2}^{13}$) for lysines and N-termini. For PICS approaches, digestion was set to semi-specific with free N-terminus, considering the respective C-terminus for tryptic and GluC-generated peptide libraries. N-terminomics data were evaluated as semi-specific cleavage with free N-terminus, taking into account the inaccessibility of dimethylated lysine for trypsin. For the PICS experiment, a protein list was obtained from the UniProt database including Swiss-Prot as well as TrEMBL entries for *Escherichia coli* K12, and this list was provided for MaxQuant searches. N-terminomics data were evaluated using solely Swiss-Prot entries for *Homo sapiens*. If not stated differently, default settings were applied for peptide searches. Decoy hits were removed from the peptide list. Subsequently, heavy-labeled peptides that had no light counterpart were considered cleaved, as well as those peptides that displayed a heavy-over-light ratio of at least 10. Cleavage sites from plasma samples were considered identified if they were consistently found in both donors investigated. Of note, PICS data were cleaned from peptides that did not show the anticipated C-terminus, i.e., C-terminal K or R for trypsin and E or D for GluC. Eventually, cleaved peptides obtained by PICS and N-terminomics workflows were analyzed with the online tool CLIP-PICS [40] and visualized using IceLogo [65]. N-terminomics data of the secretome were further processed by DAVID bioinformatics resources, employing functional annotation clustering utilizing all identified proteins as background *versus* those that were found to be processed by SpeB [66]. Proteins identified in N-terminomics of plasma were assigned to protein classes by PANTHER [67]. 

Digestion sites in IgG subclasses were manually evaluated as described earlier [24], relying mostly on GPMAW [68], ProSight [69], and Xcalibur software (version 4.2., Thermo Fisher Scientific). Isotopically resolved MS^1^ spectra were deconvoluted using the Xtract algorithm implemented in the Xcalibur software package. Unresolved MS^1^ spectra of SpeBm and SpeBz were deconvoluted with BiopharmaFinder 3.0 (Thermo Fisher Scientific), employing the ReSpect algorithm.

## Data Availability

The raw mass spectrometry proteomics data are publicly available online: https://doi.org/10.5281/zenodo.5554758 (accessed on 29 December 2021).

## Supplementary Materials

The following are available online at www.mdpi.com/article/10.3390/ijms23010412/s1.
