# Supplementary material for: Proteolytic Profiling of Streptococcal Pyrogenic Exotoxin B (SpeB) by Complementary HPLC-MS Approaches
Source: Int J Mol Sci. 2021 Dec 30;23(1):412. doi: 10.3390/ijms23010412 (PMC8745752; doi:10.3390/ijms23010412)
Supplement: Supplementary file 1 [file ijms-23-00412-s001.zip › Supplementary_figures.pdf]

Proteolytic Profiling of Streptococcal Pyrogenic Exotoxin B (SpeB) by Complementary HPLC-MS Approaches.

Constantin Blöchl [1], Christoph Holzner [2], Michela Luciano [3,4], Renate Bauer [3], Jutta Horejs-Hoeck [3,4], Ulrich Eckhard [2,5], Hans Brandstetter [2,4], and Christian G. Huber [1,4,*]

[1] Bioanalytical Research Labs, Department of Biosciences, University of Salzburg, Hellbrunner Strasse 34, Salzburg, Austria; constantin.bloechl@sbg.ac.at, c.huber@sbg.ac.at

[2] Structural Biology, Department of Biosciences, University of Salzburg, Hellbrunner Strasse 34, Salzburg, Austria; christoph.holzner@sbg.ac.at, ueccri@ibmb.csic.es, johann.brandstetter@plus.ac.at

[3] Molecular Immunology & Signal Transduction, Department of Biosciences, University of Salzburg, Hellbrunner Strasse 34, Salzburg, Austria; michela.luciano@plus.ac.at, renate.bauer@plus.ac.at, jutta.horejs-hoeck@plus.ac.at

[4] Cancer Cluster Salzburg, Department of Biosciences, University of Salzburg, Hellbrunner Strasse 34, 5020 Salzburg, Austria; michela.luciano@plus.ac.at, jutta.horejs-hoeck@plus.ac.at, johann.brandstetter@plus.ac.at, c.huber@sbg.ac.at

[5] Department of Structural Biology, Molecular Biology Institute of Barcelona, CSIC, Barcelona Science Park, Baldiri Reixac, 15-21, 08028, Barcelona, Catalonia, Spain; ueccri@ibmb.csic.es

[*] Correspondence: c.huber@sbg.ac.at

Table of Contents

Figure S1: Characterization of recombinant SpeB and protein sequence. Page S2

Figure S2: Validation of the PICS procedure employing GluC as test protease. Page S3

Figure S3: Identification of cleavage sites in proteins of the monocytic secretome. Page S4

Figure S4: Identification of cleavage sites in plasma proteins. Page S5

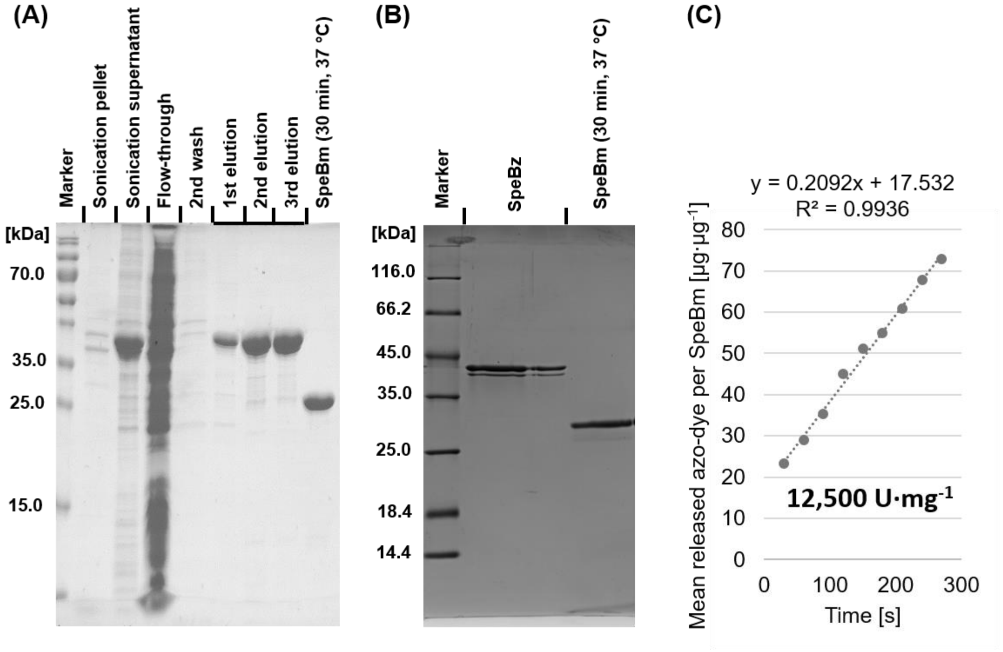

(D)

```
        10         20         30         40         50
MDQNFARNEK EAKDSAITFI QKSAAIKAGA RSAEDIKLDK VNLGGELSGS
        60         70         80         90        100
NMYVYNISTG GFVIVSGDKR SPEILGYSTS GSFDANGKEN IASFMESYVE
       110        120        130        140        150
QIKENKKLDT TYAGTAEIKQ PVVKSLLDSK GIHYNQGNPY NLLTPVIEKV
       160        170        180        190        200
KPGEQSFVGQ HAATGCVATA TAQIMKYHNY PNKGLKDYTY TLSSNNPYFN
       210        220        230        240        250
HPKNLFAAIS TRQYNWNNIL PTYSGRESNV QKMAISELMA DVGISVDMDY
       260        270        280        290        300
GPSSGSAGSS RVQRALKENF GYNQSVHQIN RSDFSKQDWE AQIDKELSQN
       310        320        330        340        350
QPVYYQGVGK VGGHAFVIDG ADGRNFYHVN WGWGGVSDGF FRLDALNPSA
       360        370        380
LGTGGGAGGF NGYQSAVVGI KPLEHHHHHH
```

Figure S1: Characterization of recombinant SpeB. (**A**) SDS-PAGE gel illustrating the purification procedure of SpeBz and its subsequent activation. Prior to SDS-PAGE analysis SpeBz and SpeBm were inhibited with MMTS (**B**) Detailed SDS-PAGE analysis of MMTS-inhibited SpeBz after purification and mature SpeBm after activation at 37 °C for 30 min. SpeBz is present as a double band: the abundant band of a proteoform of larger molecular weight corresponds to the full length SpeBz (M^{1} - H^{380}), whereas the less abundant band of lower molecular weight was found to be a cleaved proteoform (A^{28} - H^{380}). Likewise, the activated SpeBm is present as one prominent band of higher molecular weight identified as the anticipated SpeBm form (Q^{120} - H^{378}) and a broad low abundant band that is likely caused by degradation of the His6-tag (Q^{120} - H^{377} and Q^{120} - H^{376}). Annotation of species from the SDS-PAGE is based on HPLC-MS data presented in Figure 1. (**C**) Azocasein digestion by SpeBm to determine specific activity, calibrated against fully degraded azocasein. The slope of the regression equation was used to calculate the specific activity of 12,500 U·mg^{-1}. Data used to illustrate this panel is listed in Table S2. (**D**) Protein sequence of recombinantly expressed SpeBz. The pro-domain is depicted in purple whereas the sequence for SpeBm is indicated in black. In comparison to the Uniprot entry (P0C0J0), the signal peptide was removed and a C-terminal His6-tag was added. The amino acid D_2 in this construct correpsonds to the Uniprot amino acid D_{28}.

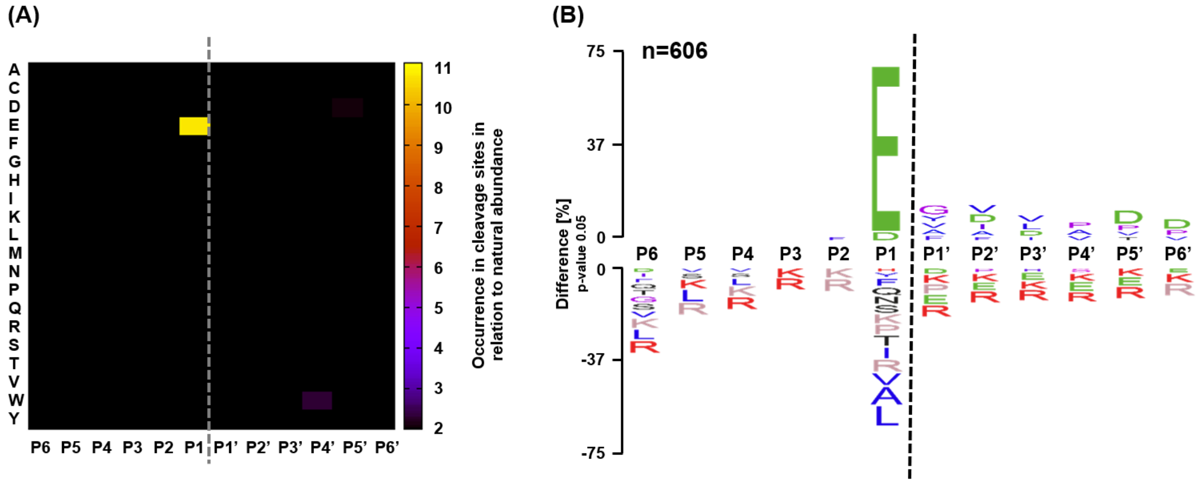

Figure S2: Validation of the PICS procedure employing GluC as test protease. 606 unique cleavage events were determined in a tryptic peptide library employing HPLC-MS analysis. (**A**) Heatmap depicting the fold-change in the occurrence of specific amino acids at certain positions in the substrate in relation to their natural abundance. (**B**) Sequence logo demonstrating the difference in percent of amino acids at specific positions in prime and non-prime sites employing the IceLogo algorithm. The color code represents the physico-chemical properties of amino acids: hydrophobic residues (blue), positively charged residues (red), negatively charged residues (green), hydrophilic (black) and others (pink). Residues that were not found in any cleaved peptides at a specific position are depicted in grey. The underrepresenations of K and R is a consequence of the tryptic library.

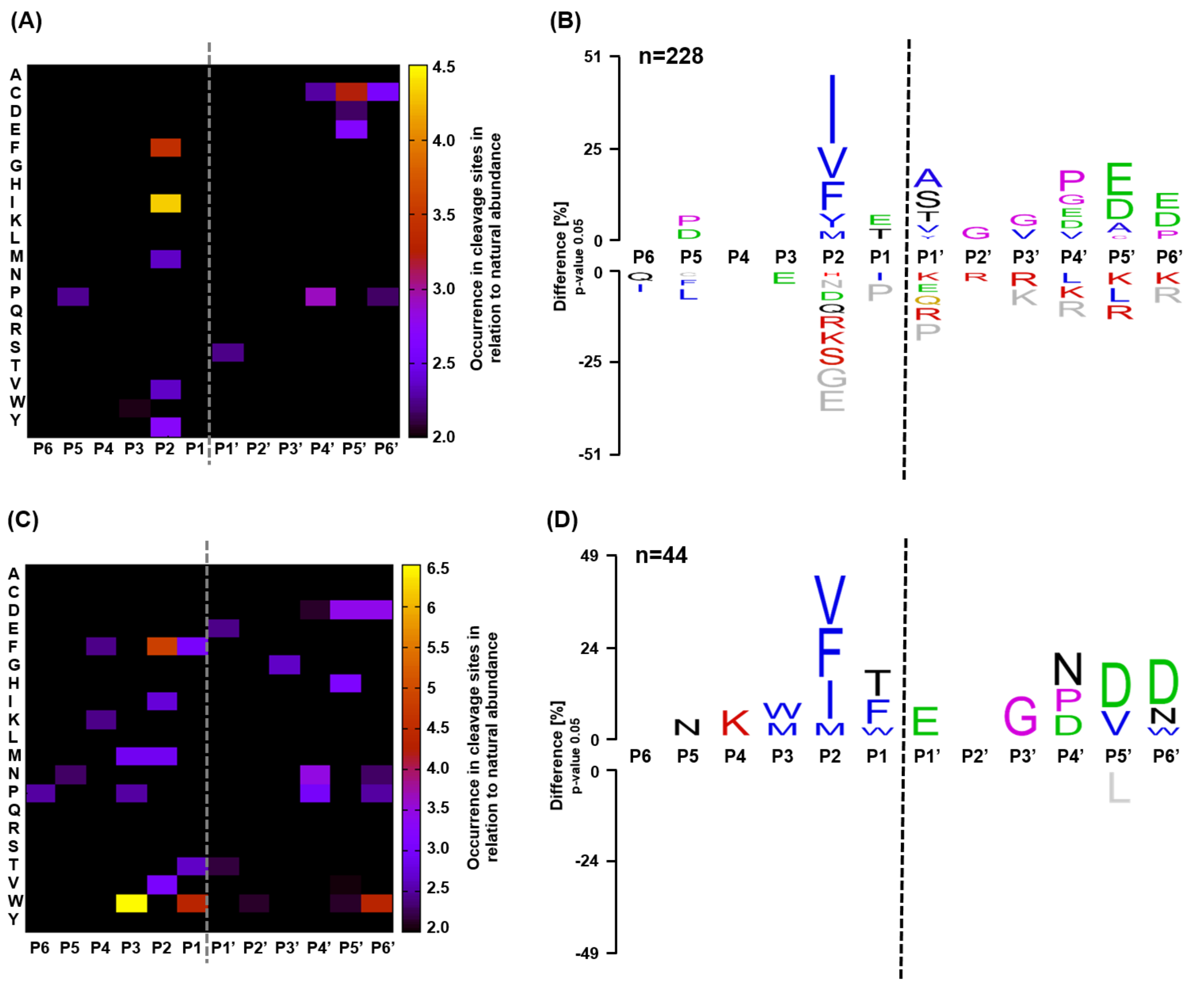

Figure S3. Identification of cleavage sites of SpeBm in intact proteins derived from the monocytic secretome. 272 cleavage sites were identified by the N-terminomics workflow combining peptides identified after subsequent tryptic (**A**, **B**) and GluC digestion (**C**, **D**), respectively. Heatmaps in panel **A** and **C** depict the fold-change in occurrence of specific amino acids at certain positions in the substrate in relation to their natural abundance. In the panels **B** (tryptic) and **D** (GluC) the respective sequence logos obtained by IceLogo analysis are depicted. These amino acid logos are demonstrating the difference in perecentage of amino acids at specific positions in prime and non-prime sites ranging from P6 to P6´. The color code represents the physico-chemical properties of amino acids: hydrophobic residues (blue), positively charged residues (red), negatively charged residues (green), hydrophilic (black), and others (pink). Residues that were not found in any cleaved peptides at a specific position are depicted in grey.

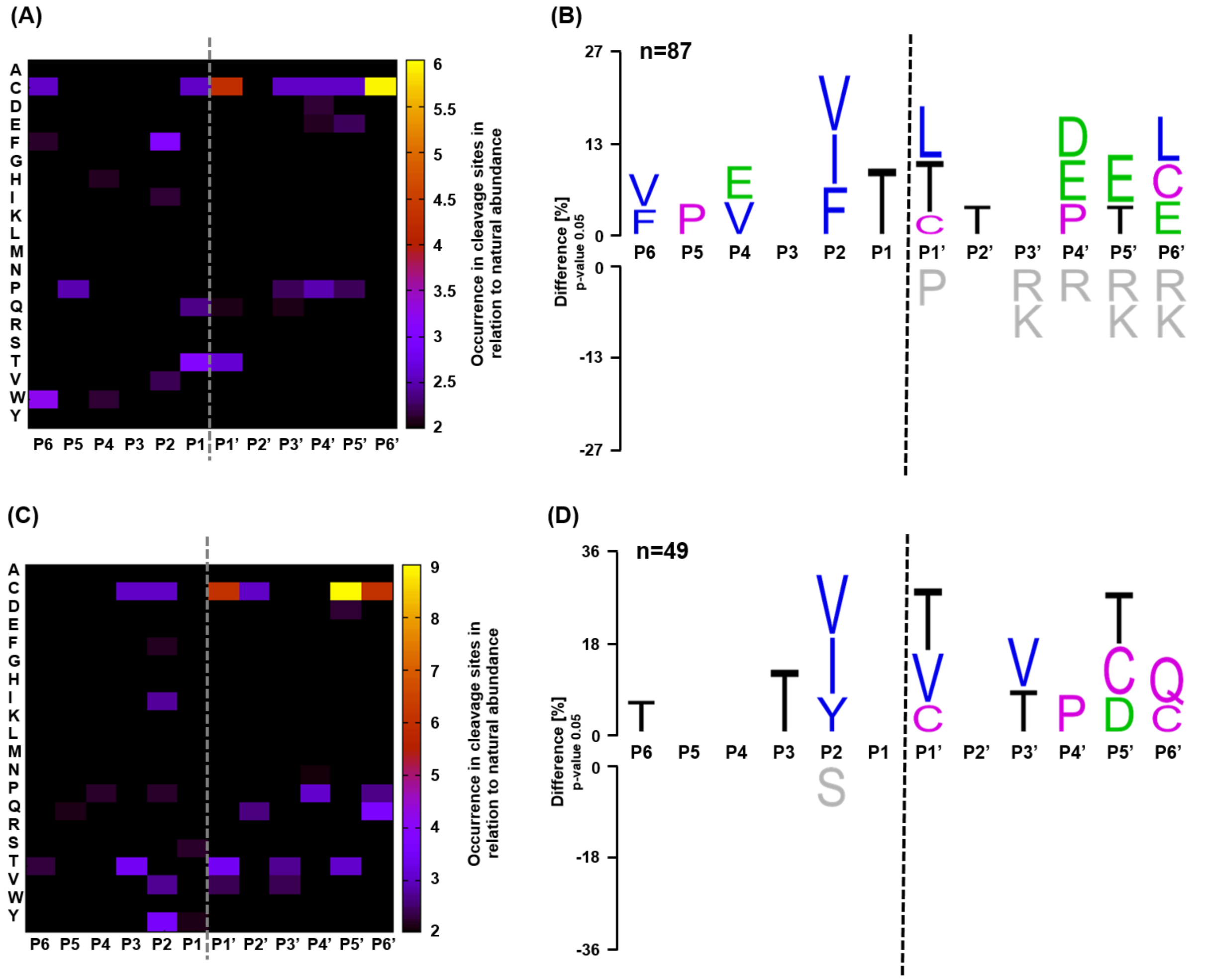

Figure S4. Identification of cleavage sites of SpeBm in intact plasma proteins. 136 cleavage sites were identified by the N-terminomics workflow combining peptides identified after subsequent tryptic (**A**, **B**) and GluC digestion (**C**, **D**), respectively. Heatmaps in panels **A** and **C** depict the fold-change in occurrence of specific amino acids at certain positions in the substrate in relation to their natural abundance. In the panels **B** (tryptic) and **D** (GluC) the respective sequence logos obtained by IceLogo analysis are depicted. These amino acid logos are demonstrating the difference in percent of amino acids at specific positions in prime and non-prime sites ranging from P6 to P6´. The color code represents the physico-chemical properties of amino acids: hydrophobic residues (blue), positively charged residues (red), negatively charged residues (green), hydrophilic (black), and others (pink). Residues that were not found in any cleaved peptides at a specific position are depicted in grey.
